# Editorial: Beyond Embodied Cognition: Intentionality, Affordance, and Environmental Adaptation

**DOI:** 10.3389/fpsyg.2018.02659

**Published:** 2018-12-21

**Authors:** Zheng Jin, Maurizio Tirassa, Anna M. Borghi

**Affiliations:** ^1^International Joint Laboratory of Behavior and Cognitive Science, Zhengzhou Normal University, Zhengzhou, China; ^2^Department of Psychology, University of Turin, Turin, Italy; ^3^Department of Dynamic and Clinical Psychology, Sapienza University of Rome, Rome, Italy; ^4^Institute of Cognitive Sciences and Technologies, Italian National Research Council, Rome, Italy

**Keywords:** embodied cognition, intentionality, affordance, environmental adaptation, ecology

Considering that humans must use external tools to solve problems, any account of human cognition should incorporate such intentional tool-using processes into its models of environmental adaptation. In the traditional ecological paradigm and embodied cognitive science, affordances (i.e., possibilities for action which are available for an agent to perceive directly and act upon) are nested in environment (Gibson, 1979). Intentionality is defined as a power of minds that simultaneously coordinates with multiple affordances (e.g., Kiverstein and Rietveld, 2015). Exploring potential mechanisms that are responsible for intentionality would open up new avenues for developing alternative paradigms of psychology on differing assumptions regarding the relationship among mind, body, and environment. This Research Topic is devoted to the particular question; how embodied cognitive processes contribute to the adaptation to a given environment with intentionality.

One of the interests of this Research Topic was to provide an explanation of the relationship between the sensorimotor process and one's interaction with the environment. Huang et al. aimed at the exploring the potential dissociation between the sense of 1PP-location (i.e., first-person perspective) and body-location. In doing so they approached a topic that is of great interest in the field of self-consciousness and self-perception. Since the sense of self-location is crucial for one's interaction with the environment, recognizing the distinctive roles of 1PP-location and body-location would contribute to a better picture of environmental adaptation. Their data showed that under different manipulations of movement, the spatial unity between 1PP-location and body-location could be temporarily interrupted. Interestingly, they also observed a “double-body effect” and further suggested that it is better to consider body-location and 1PP-location as interrelated but distinct factors that jointly support the sense of self-location. Their conclusion may help to explain the tremendous flexibility of our bodily experiences in coping with novel environmental challenges. By recruiting patients with schizophrenia, Sevos et al. examined whether the addition of a more salient action context can promote the emergence of affordance effect during the perception of everyday objects. Participants performed two Stimulus–Response-Compatibility tasks in which they were presented with semantic primes related to sense of property or goal of action prior to viewing each graspable object. Controls responded faster when their response hand and the graspable part of the object were compatibly oriented, but only when the context was congruent with the individual's needs and goals. When the context operated as a constraint, the affordance-effect was disrupted. These results support the understanding that object-affordance is flexible and not just intrinsic to an object. The authors also noted that the lack of sensorimotor facilitation in patients with schizophrenia would require extensive use of higher cognitive processes even for the simplest routine activities in their daily life. Their conclusion was informative to understand the specific mechanisms behind schizophrenia.

Another appealing questions in this topic is how affordances are perceived. Human infants are not born understanding how to perceive affordance. It takes two corequisite sets of control processes to explain the manner of the affordances learning (Iran-Nejad and Bordbar). For conceptual understanding (CU), knowers have deliberate attention-allocation control over their first-person “knowthat” and “knowhow” content combined as mutually coherent corequisites. For biofunctional understanding (BU), knowers have attention-allocation control only over their knowthat content but knowhow control content is ordinarily conspicuously absent. With a thematic focus on embodiment science and an eye toward systematic consensus in systemic cohesion, Iran-Nejad and Bordbar's study explored the roles of biofunctional and conceptual control processes in the wholetheme spiral of biofunctional understanding. They tested a hypothesis of the difference between CU and BU. Their findings supported the notion that individuals are capable of engaging in mind-body cohesion-sensing and consensus-seeking practices. These findings are also discussed in terms of the predicted differences between BU and CU control processes, their roles in regulating the physically unobservable flow of systemic cohesion in the wholetheme spiral, and a proposal for systematic consensus in systemic cohesion to serve as the second guiding principle in biofunctional embodiment science next to physical science's first guiding principle of systematic observation. Ramstead et al. extended the notion of affordance to encompass the sociocultural level and the scaffolding it provides to cognition. They investigated the ways in which people perceive and engage with cultural affordances. Aiming to account for the relationship between cultural content and normative practices on the one hand and immersive participation on the other hand, they focused on the social practices that regulate joint attention and shared intentionality.

We received a study from Schilhab noticing that the multi-functional nature of smart technology leading to noticeable changes in affordances and embodiment. She addressed the question to how social interaction (e.g., deep conversations) facilitates the development of offline-cognition (e.g., mental imagery, stream of consciousness, etc.) that enables the (self-) regulation of online cognition and interactions with technology. This opinion article took us back to 2002, when one of Wilson's much-cited six claims were published (Wilson, 2002). To some extent, this article, together with Lee et al. (2012)'s results, again invites us to take more seriously the philosophical issue of the “natural kind” (e.g., Millikan, 1999; Ellis, 2001) for which off-line aspects of embodied cognition is a proxy in on-line interactions with the environment. In traditionally view, Behavior is thought to be a means to control the environment, which ignores the fact that the target object can be perceived through activity. For example, some people take their seat after confirming the space between the table and the chair; others first sit down and then re-evaluate or adjust the distance. Therefore, even though the initial stimulus is always perception, the interaction continues back and forth between perception and action. Cognition serves to guide the behaviors that acquire perceptions needed for new behaviors. The two behaviors, before or after reappraisal, communicate information. Gibson (1979) conceptualized this information as survival-related symbols given by the environment to an organism. Shaw extended this definition with the concept of intention (e.g., Shaw, 2001). In brief, to survive, organisms coordinate with their environment, communicate information, and realize intentions. The conceptualized living (or survival) is similar to the Gih of oriental philosophy. In this Topic, Lee et al. attempts to build a meta-theory and to demonstrate empirical designs for Gih, discussing the problems of the mind and body, or the subject and object, compared with the concept of “affordance” proposed by ecological approaches. They claimed that Gih should not remain in the domain of mysticism; the concept may be addressed by psychological science to make use of valuable insights from Eastern philosophy through empirical research.

Three studies investigated whether and how one important characteristic of the sensory, motor, and emotional system is reflected in language processing. Marino et al. report two experiments on the relationship between language and affordances. Participants were presented with short sentences composed by verbs referring to motor chains and nouns of tools, and were required to decide whether the image following the sentence was mentioned in it or not. The results showed that the grasp verb motor chain activated volumetric information, while the functional motor chain activated information related to tool use. Overall the studies demonstrate the influence of the motor system and of its chained organization on language processing. Buccino et al. investigated the embodiment of second language and evidenced that embodied cognitive processes appear to be substantially the same in L2 as it is in L1. Starting from the available evidence to the effect that language processing relies on the same sensory, motor, and emotional structures that are involved when individuals experience the contents of language material, they found that the processing of English nouns by native speakers of Italian who also speak English recruits the same neural substrates as the Italian equivalents. Baumeister et al. investigates whether the link between language and emotion is reduced in L2. Late Spanish-English bilinguals were required to categorize a set of English and Spanish words into “associated to emotion” or “not associated to emotion,” then they were submitted to a surprise recognition task (old/new word). Electromyography (EMG) and skin conductance (SC) were recorded; in particular, muscle activity for corrugator and zygomaticus muscles in response to happy and angry emotional words for both L1 and L2 was detected. Results indicate stronger enhancement of memory for emotional over neutral stimuli in L1 than in L2; furthermore, results of the EMG and SC recordings indicate a slightly reduction of facial motor resonance and SC responses to emotional stimuli in L2. In line with embodied cognition views, they suggest that the processing of emotional L2 words is less grounded in the motor, sensory, and autonomic nervous systems than the processing of L1 words.

In addition, one general original research and one commentary article were concerned with the methodological issues on embodied cognition research. Cantarero et al. focus on the relationship between gestures and moral behavior and investigates whether body gestures commonly associated with (dis)honesty influence white lies. Participants were asked to give feedback about the work of an artist they did not like in his face, facing the dilemma between telling him the truth or lying to him, thus preserving him from feeling bad (other oriented lie). During the conversation they had to hold the hand-over-heart gesture, typically related to honesty, or the fingers crossed, and hand over elbow gestures. In the first experiment they find that the hand-over-heart gestures is less associated to other oriented lies. In the pre-registered experiment 2 they did not replicate the previous result: the hand-over-heart gesture did not impede participants to use other-oriented white lies. The authors discuss their results in the framework of research on embodied cognition, arguing that high methodological standards are necessary, in particular when effect sizes are small. Based on Sevos et al. data, Faulkenberry and Tummolini's commentary pointed out the issues that are present when trying to interpret non-significant results in the traditional null hypothesis statistical testing framework, and offered a quick example of how to use a Bayesian approach to quantify evidence for object-affordance effects and other action-specific influences on perception in the study of embodied cognition.

This topic also comprises articles from other distinctive perspectives which speak to the multifaceted research in this field. Einarsson and Ziemke's contribute to this research topic, is an illustration—using the case of interactive music—of how seemingly highly abstract, disembodied and unsituated activities, such as the composition of musical works, can in fact be strongly grounded in concrete embodied and situated activity. Their theoretical perspectives and concrete examples may help to elucidate how situations—and with them affordances—are dynamically constructed through the interactions of biological, contextual, social, and cultural mechanisms as embodied and situated activity unfolds. Martínez-Pernía et al. introduced a level of treatment that precedes behavior and cognition in a case study. This theoretical consideration allowed the discovery of a better relation between affordance and the environmental adaptation for the improvement behavioral and cognitive performance in their case study.

The final collection of 13 articles provides an overview of current trends and opinions, as well as perspectives on theoretical and methodological questions. As pointed out by our CFP, psychology has continued to formulate and refine a variety of paradigms to provide solutions for the mind-body problem. Although a number of contemporary psychologists believe they have avoided dualism by noting the close relationship between certain brain activities and certain cognitive events, it appears likely that such a relationship will soon be discovered for all mental events. Replacing the term mind-body with the term mind-brain does little to solve the problem of how the brain can cause something mental. The traditional metaphysics founded in subject-object dichotomy is still at the basis of the majority of paradigms in psychology. We hope that the reader will find the collected articles both informative and thought-provoking, and that this Research Topic will stimulate the scientific debate contributing to overcome such a dichotomy.

## Author Contributions

ZJ drafted the paper. All authors provided critical comments and additions, and approved it for publication.

### Conflict of Interest Statement

The authors declare that the research was conducted in the absence of any commercial or financial relationships that could be construed as a potential conflict of interest.

## References

[B1] Ellis B. (2001). Scientific Essentialism. Cambridge: Cambridge University Press^®^

[B2] Gibson J. J. (1979). The Ecological Approach to Visual Perception. Boston, MA: Houghton Mifflin^®^

[B3] Kiverstein J. Rietveld E. (2015). The primacy of skilled intentionality: on Hutto & Satne's the natural origins of content. Philosophia 43, 701–721. 10.1007/s11406-015-9645-z 30158720 PMC6100026

[B4] Lee Y Lee S. Carello C. Turvey M. T. (2012). An archer's perceived form scales the “hitableness” of archery targets. J. Exp. Psychol. Hum. Percept. Perform. 38:1125. 10.1037/a0029036 22731994

[B5] Millikan R. G. (1999). Historical kinds and the “special sciences”. Philos. Stud. 95, 45–65.^®^

[B6] Shaw R. (2001). Processes, acts, and experiences: three stances on the problem of intentionality. Ecol. Psychol. 13, 275–314. 10.1207/S15326969ECO1304_02^®^

[B7] Wilson M. (2002). Six views of embodied cognition. Psychon. Bull. Rev. 9, 625–636. 10.3758/BF03196322 12613670

